# Effect of Propolis on Diet-Induced Hyperlipidemia and Atherogenic Indices in Mice

**DOI:** 10.3390/antiox8060156

**Published:** 2019-06-03

**Authors:** Nada Oršolić, Irena Landeka Jurčević, Domagoj Đikić, Dunja Rogić, Dyana Odeh, Vedran Balta, Eleonora Perak Junaković, Svjetlana Terzić, David Jutrić

**Affiliations:** 1 Division of Animal Physiology, Faculty of Science, University of Zagreb, Rooseveltov trg 6, HR-10000 Zagreb, Croatia; domagoj.djikic@biol.pmf.hr (D.Đ.); dyana.odeh@biol.pmf.hr (D.O.); vedran.balta25@gmail.com (V.B.); 2 Laboratory of Chemistry and Food Biochemistry, Faculty of Food Technology and Biotechnology, University of Zagreb, Pierottijeva 6, 10000 Zagreb, Croatia; ilandeka@pbf.hr; 3 Department of Medical Biochemistry and Hematology, Faculty of Pharmacy and Biochemistry, University of Zagreb, Domagojeva 2, 10000 Zagreb, Croatia; dunjarogic@hotmail.com; 4 Laboratory for Veterinary-Medicinal Products, Croatian Veterinary Institute, Savska cesta 143, 10000 Zagreb, Croatia; perak@veinst.hr (E.P.J.); terzic@veinst.hr (S.T.); 5 Clinical Hospital Dubrava, Avenija Gojka Šuška 6, 10000 Zagreb, Croatia; davidjut2000@yahoo.com

**Keywords:** high-fat diet, propolis, triglycerides, HDL and LDL-cholesterol, atherogenic indices, antioxidative capacity

## Abstract

Obesity, a major health problem worldwide, is associated with increased cardiovascular risk factors, such as dyslipidemia, glucose intolerance, and hypertension. We investigated the antioxidative capacity of the ethanol extract of propolis (EEP) and its effect on the lipid profile, the hepatorenal function, and the atherogenic indices in mice fed with a high-fat diet (HFD). EEP (50 mg/kg) was given orally to mice for 30 days. After the treatments, levels of the serum total triglyceride and cholesterol, the high density lipoprotein (HDL-c) and low density lipoprotein (LDL-c) cholesterols, the serum enzymes, and the metabolites were measured, and atherogenic indices [atherogenic index of plasma (AIP); cardiac risk ratio (CRR); cardioprotective index (CPI); atherogenic coefficient (AC)] were calculated and compared with the antioxidant, the reducing power, the radical-scavenging, and the chelating activity of EEP. The HFD diet with EEP significantly reduced the negative lipid profile and lowered AIP, CRR, and AC and increased CPI in animals on a HFD. In addition, EEP reduced the weight of mice and lipid accumulation in the liver, and it had significant in vitro antioxidative activities. The EEP possesses anti-hyperlipidemic and antioxidant activity and exhibits protective action on the cardiovascular system and hepatorenal functions. Our results contribute towards the validation of the traditional use of propolis as a food supplement in aiding hyperlipidemic disorders.

## 1. Introduction

The basic cause of obesity is the imbalance between energy intake and energy expenditure. Accumulation of fat tissue, especially the accumulation of visceral fat tissue, is a possible primary cause of “metabolic syndrome”, which is the combination of multiple cardiovascular risk factors such as dyslipidemia, glucose intolerance, and hypertension. A number of epidemiological studies have reported positive associations between fat intake, level of blood cholesterol, and risk of coronary heart disease (CHD) and CHD mortality. Dyslipidemia and oxidative stress, which are closely related to diabetes, hypertension, obesity [1,2,3,4], and cancer [5], might be consequences and causes of the high mortality of humans and animals from cardiovascular diseases [5,6]. Nutritional and pharmacological preparations that affect such physiological conditions, which accompany dyslipidemia, elevated plasma triglyceride levels, total low density lipoprotein (LDL) and very-low-density lipoprotein (VLDL) cholesterol, and a low level of high density lipoprotein (HDL) cholesterol, can regulate lipid metabolism and be effective in reducing the risk of lipid disorders, obesity, cardiovascular diseases, and consequent pathological complications [7]. Recently, there has been renewed interest in the use of herbal bee products in the preventive treatment of many different chronic diseases, such as osteoporosis, cancer, psoriasis, and diabetes [4,5,6,7,8,9,10,11]. The advantages of using natural preparations are easy availability, better usability, lack of adverse effects, and economic cost-effectiveness. Propolis has been greatly popular as an agent in traditional medicine and as a food dietary supplement for human health in the world [12,13]. Propolis is produced by honeybees using collected extracts from leaves, buds, and exudates of various plant floras [14]. Its antibacterial, antiviral, antitumor, anti-inflammatory, anticancer, antiallergic, antilipidemic, and vasodilatory actions, as well as its hepatoprotective and immunomodulatory effects, have been reported [13,15,16]. In addition, flavonoids from propolis can inhibit lipid peroxidation, platelet aggregation, capillary permeability and fragility, and the activity of enzyme systems including cyclooxygenase and lipooxygenase [5,8,10]. More than 300 molecular species, such as alkaloids, aromatic acids and their esters, free acids and their esters, phenolic acids and their esters, phenolic aldehydes, alcohols and ketones, sesquiterpenes, quinones, flavonoids, minerals, and vitamins are isolated from propolis [17,18], and their ratios and concentration depend on geographical origin. Flavonoids and phenolic compounds have strong antioxidant properties and the ability to protect cells from reactive radicals and protect important biological molecules, such as lipids, proteins, and DNA. Flavonoids can be aglycones, glycosides, and methylated derivatives, and their antioxidant activity depends on the arrangement of functional groups around the nuclear structure—in particular, the total number of hydroxyl groups [17,18]. For example, the hydroxyl configuration of the B ring is important for the removal of reactive oxygen species (ROS) and reactive nitrogen species (RNS) due to hydrogen and electron donation to hydroxyl, peroxyl, and peroxynitrite radicals, which stabilizes them and produces stable flavonoid radicals. It is believed that aglycones are more potent antioxidants than the corresponding glycosides, and that the degree of polymerization increases the antioxidant capacity, which is important for their biological and pharmacological efficacy [3,5,19].

In spite of the multitude of ethnomedical applications against various pathophysiological conditions, we found scarce data in literature relating to the effect of propolis on obesity, plasma lipid profiles, and atherogenicity in a high-fat diet-induced obesity mouse model. In this connection, we conducted a study to find out whether propolis could reduce dyslipidemia induced by the intake of high-fat diet and thus protect the liver function and cardiovascular system. The effect of propolis on cardiovascular function was assessed by measuring lipid profile parameters that we used to calculate atherogenic indices, namely the atherogenic index of plasma (AIP), the cardiac risk ratio (CRR), and the atherogenic coefficient (AC). Due to the numerous antioxidant mechanisms of flavonoids present in propolis, including the free radical scavenging capacity, the inhibition of prooxidative enzymes, the metal chelating activity, and the synthesis of antioxidative enzymes (useful in protecting the body from hypertension, atherosclerosis, and cardiovascular diseases), we believe that it is worth investigating the effect of propolis on dyslipidemia induced by the intake of a high-fat diet. The goal is to try to prevent the consequences of dyslipidemia, e.g., cardiovascular diseases and hepatorenal disturbances.

## 2. Materials and Methods

### 2.1. Reagents and Instruments

All chemicals to investigate the lipidemic properties, the antioxidant capacity, and the analytics of propolis were purchased from Sigma-Aldrich Chemical Co. (St. Louis, MO, USA) except ethanol, methanol, potassium acetate, potassium hydroxide, and sodium carbonate, which were purchased from Kemika (Zagreb, Croatia) or from Merck, Darmstadt, Germany (AlCl_3_ and 2,4-dinitrophenylydrazine). Perkin Elmer Lambda 25 spectrophotometer (Perkin Elmer, Waltham, MA, USA) was used for the absorbance measurements.

### 2.2. Animals

Experiments were carried out according to the guidelines enforced in Croatia (Law on the Welfare of Animals, NN 19/1999) in accordance with the internationally accepted principles for laboratory animal use and care as found in the European Community guidelines (EEC Directive of 1986; 86/609/EEC). The ethical committee (Faculty of Science, University of Zagreb, Croatia) approved the present study (approval code: URBROJ: 251-58-10617-13-57). Inbred male C57BL/6N (60 ± 5 days) weighing ~24 g from the mouse colony of Faculty of Science, University of Zagreb were used. The animals were kept under a 12 h light, 12 h dark light-dark regime at 60% humidity. The animals were maintained on a formulated commercial pelleted diet, and water was provided ad libitum. Food pellets were a certificated standard mice and rat diet 4RF21 (Mucedola, Settimo Milanese, Italy; Batch No. 238603, shape 12 mm). The composition of the standardized pellet mouse feed is shown in Table 1.

### 2.3. Ethanol Extract of Propolis (EEP)

Propolis was collected by scraping it off from hive frames in the outskirts of Zagreb, Croatia and kept in the dark at room temperature until analysis. It was prepared according to the method described in the paper [20]. Propolis (10 g) was crushed, extracted in ethanol for 48 h, filtered through Whatman Grade 4 paper (Sigma Aldrich Chemical Co., St. Louis, MO, USA), and then the extract was lyophilized. The ethanol extract of propolis (EEP) was dissolved in ethanol, and after that, in water. The final concentration of ethanol was up to 1%. The control group was treated with the same solution.

### 2.4. Total Phenol Content (TP), Total Flavonoid Content (TF), and Total Phenolic Acid Content (TPA)

We used spectrophotometric procedures for quantification of the three main groups of bioactive substances in propolis [total phenol content (TP); total flavonoid content (TF); total phenolic acid content (TPA)] that were important for the evaluation of the propolis biological activity [21]. The total phenol content in the extracts was determined by the Folin–Ciocalteau colorimetric method as in Singleton et al. [22]. The total flavonoid content was determined by the method of Kumazawa et al. [23]. The total phenol content was expressed as gallic acid equivalents (mg/g), and the total flavonoid content as quercetin equivalents (mg/g) from calibration curves recorded from the standards. The total phenolic acid content was calculated according to European Pharmacopoeia (Ph. Eur.) as rosmarinic acid equivalents (mg/g).

### 2.5. High-Performance Liquid Chromatography (HPLC) Analysis of Ethanol Extract of Propolis EEP

HPLC analysis of EEP was performed according to the method described by Pietta et al. [24] and also described in paper [19]. For EEP analysis, we used the Shimadzu AV system with a UV-visible detector (model SPD-10A, Shimadzu Co., Tokyo, Japan) and a VP-class software (version 6.13 SP2, Shimadzu Co., Tokyo, Japan). Diluted EEP [1:50 (*v*/*v*) with methanol (Merck, Darmstadt, Germany)] was injected into the C_18_-column 100 × 0.2 mm with 5 μm particle size (Bischoff Chromatography, Leonberg, Germany). The flow rate was set to 1.5 mL/min at an oven temperature of 30 ℃, and separations were performed by elution of columns by using a linear gradient of acetonitrile (A) (LiChrosolv^®^, Merck, Darmstadt, Germany) and 0.2% (*v*/*v*) acetic acid (B) (J.T. Baker Chemical Co., Phillipsburg, New Jersey, USA) in ultra-pure water (Millipore, Watford, United Kingdom) with gradients: 10–40% (B) in 40 min, 40–55% (B) in 10 min, and decreased (B) to 10% in 10 min. The chromatograms were recorded at 290 nm, and compounds were identified and quantified by standards (quercetine-dihydrate, chrysin, galangin, naringenin, and caffeic acid, Sigma-Aldrich) previously isolated from the poplar-type of propolis. Standards (2 mg in 10 mL) and working solutions were prepared in methanol to obtain five calibration concentrations in a range between 2 and 200 mg/mL with *r*^2^ > 0.995. All solvents used were of HPLC-grade.

### 2.6. Antioxidant Capacity

EEP antioxidant activity was assessed using four different assays in vitro: 2,2-diphenyl-1-picrylhydrazyl radical-scavenging (DPPH), reducing power, chelating activity, and β-carotene bleaching assays (BCB). Each test was done with three replicates.

### 2.7. β-Carotene Bleaching Assay

The antioxidant activity of the EEP was evaluated using the β-carotene-linoleic acid system according to Amarowicz et al. [25]. In the BCB assay, linoleic acid was converted to the linoleic free radical, which reacted with β-carotene and caused its degradation. Antioxidants reacted with free radicals present in the solution, reducing the rate of reaction. The bleaching rates of the solution with and without the antioxidant were calculated as antioxidant activity (ANT):

ANT = (R_control_ − R_sample_)/R_control_ × 100 (1)

where R_control_ and R_sample_ are the average bleaching rates of water control and the antioxidant [plant extract or butylated hydroxyanisole (BHA)], respectively.

### 2.8. DPPH Radical-Scavenging Activity

Radical scavenging activity (RSA) was evaluated using DPPH free radicals described in [26]. The mixtures of 1.0 mL of 0.16 mM DPPH methanolic solution with 1.0 mL of either methanolic solution of extract (sample) or methanol (control) were vortexed, stranded at room temperature in the dark for 30 min, and the absorbance was read at 517 nm. The RSA for DPPH free radical was calculated as:

RSA = (A_control_ − A_sample_)/A_control_ × 100 (2)

where A_control_ and A_sample_ are the absorbance. The concentration of the sample that inhibits 50% of the DPPH free radical was calculated from the graph of RSA percentage against extract concentration. BHA was used as the antioxidant standards for comparison of the activity.

### 2.9. The Reducing Power of the Extracts

The ability of the ethanolic extract to donate an electron was assessed by using the reducing power assay method of Yen and Chen [27]. The method is based on the reaction mixture of extract (0.2–1 mg), potassium ferricyanide (Fe^3+^), and sodium phosphate buffer. After incubation at 50 ℃ for 30 min, trichloroacetic acid was added, and the mixture centrifuged. The supernatant was recovered, mixed with deionized water and ferric chloride, and the absorption was measured at 700 nm. Ascorbic acid at various concentrations was used as standard. The details of the method are described in [28]. Increased absorbance of the reaction mixture indicated an increase in the reducing power. Reducing power was expressed as a slope of the trendline (SRP) of absorbance of the reaction mixture versus the concentration of EEP or ascorbic acid.

### 2.10. Chelating Activity (ChA)

Metal chelating activity (ChA) was studied using ferrozine and Fe^2+^ ions, and the absorbance was read at 562 nm [28]. The various concentrations of the extracts were incubated with 0.05 mL of FeCl_2_·4H_2_O (2 mM), and the reaction was initiated by the adding 5 mM ferrozine (0.2 mL). After 10 min, the absorbance was read at 562 nm. The percentage of chelating ability was determined by the formula:

ANT = (A_control_ − A_sample_)/A_control_ × 100 (3)

where A_control_ is the absorbance of the negative control (solution to which no extract was added), and A_sample_ is the absorbance of the extract solution. Chelating activity was expressed as ChEC_50_, the concentration that chelates 50% of Fe^2+^ ions and thus has ChA = 50%.

## 3. Experimental Design

For the treatment group, edible sunflower seed oil (Zvijezda, Zagreb, Croatia) was used as the high-fat diet (HFD). A ratio of unsaturated/saturated = 7.3, 7% palmitic acid, 5% stearic acid, 19% oleic acid, 68% linoleic and 1% alpha linolenic acid was used. Through the whole experiment, on the days of treatment, the body weights of the animals were recorded, and food intake was monitored every two days. Each group contained nine animals. The first group was the control group treated with 0.2 mL/d of water with 1% of ethanol (vehicle). The second group was given the ethanol extract of propolis (EEP) (0.2 mL/d) at a dose of 50 mg/kg [3]. The third group was treated with HFD (sunflowers seed oil, 0.1 mL/d) together with 0.1 mL/d of vehicle. The fourth group received the same amount (0.1 mL/d) of sunflower seed oil together with the ethanol extract of propolis (0.1 mL/d) at a dose of 50 mg/kg (HFD + EEP). All treated groups received (by gauge) a total volume of 0.2 mL/d repeatedly for 30 days. To avoid the false changes influenced by excessive blood loss caused by the frequent sampling, the whole blood samples and liver were collected on the 30th day of the experiment, respectively. On the 30th day, the animals were anesthetized (isoflurane, 2% in a flow of oxygen) and exsanguinated by intra-cardiac puncture without anticoagulant, as described in European Mouse Phenotyping Resource for Standardized Screens (EMPReSS), SOP (standard operating procedure) [29] under veterinary supervision. After coagulation, the un-hemolyzed serum was collected and frozen at −80 ℃ until further processing. The liver was isolated from the animals instantly, and all further processing was done on ice. The liver weight was recorded. Livers were isolated and frozen at −80 ℃ until further processing. A sample of the whole liver (15–25 mg) was taken and processed for further triglyceride and total cholesterol assay protocols.

The whole experiment was repeated twice, and the statistical analysis showed no difference between the first and the second experimental setup (repetition).

### 3.1. Body Weight

During the study period of 30 days, the mice were weighed every day using the electronic balance, and their body weights were recorded. The body weight was expressed as a difference between the body weight on a certain day minus the weight on the initial day.

### 3.2. Estimation of the Serum Liver Enzyme Activity and Kidney Function

Serum biochemical parameters for renal and hepatic function included aspartate aminotransferase (AST), alanine aminotransferase (ALT), alkaline phosphatase (ALP), urea, creatinine, blood glucose levels (glucose), lactate dehydrogenase (LDH), and total protein. After coagulation, the un-hemolyzed serum was collected and frozen at −80 ℃ until the further processing of biochemical parameters with the apparatus Alcylon 300 (Abbott, Lake Forest, IL, USA). The percentage of glycemic changes was calculated according to the formula:

Glycemic changes (%) = (G_x_ − G_0_)/G_0_ × 100 (4)

where G_0_ = blood glucose level at day zero, and G_x_ = blood glucose level at day 30.

### 3.3. Estimation of Lipid Profile Parameters in Serum and Liver

The experiment and the biochemistry analysis were conducted according to the recommendations of the International Federation of Clinical Chemistry (IFCC) methods in enzymology and were done with commercial kits (Merck, Darmstadt, Germany) on the Hitachi 717 automatic analyzer (Hitachi, Tokyo, Japan). All analyzed parameters were measured from the un-hemolyzed blood serum and liver homogenate at room temperature. Briefly, the activity of LDH (E.C.1.1.1.27) was measured under 340 nm by pyruvate to lactate continuous turnover measurement reaction [30]. ALP (E.C.3.1.3.1.) was measured at 405 nm, substrate 4-nitrophenilphosphate [31]. AST (E.C. 2.6.1.1.) and ALT (E.C.2.6.1.2.) were measured at 340 nm [32]. Creatinine concentrations were measured with the method of alkaline picrate complex at 492 nm [33]. Total protein concentration was measured by the method of Lowry [34]. Triglycerides were measured by the colorimetric enzymatic method, which utilized the lipoprotein lipase for rapid and complete hydrolysis of triglycerides into glycerol followed by oxidation to dihydroxyacetone phosphate and hydrogen peroxide. The hydrogen peroxide reacted with 4-aminophenone and 4-chlorophenol under peroxidase catalytic action to form a red dye. The concentration of triglycerides was proportional to the intensity of the color generated and measured photometrically.

Total cholesterol was evaluated by enzymatic colorimetric method. Cholesterol esters were cleaved through the action of cholesterol esterase, producing free cholesterol and fatty acids. Cholesterol oxidase catalyzed the oxidation of cholesterol to cholest-4-en-3-one and hydrogen peroxide. In the presence of oxidase, the formed hydrogen peroxide affected the oxidative coupling of phenol and 4-aminoantipyrine, forming a quinone-imine red dye. The color intensity was directly proportional to cholesterol concentration, and the absorbance reading was at 512 nm.

High density lipoprotein cholesterol (HDL-c) was analyzed by the homogeneous colorimetric enzymatic method. In the presence of magnesium ions, dextran sulfate selectively formed water soluble compounds with low density lipoprotein cholesterols (LDL-c), very-low-density lipoprotein cholesterol (VLDL-c), and chylomicrons, which are resistant to polyethylene glycol-modified enzymes. Under the influence of the cholesterol enzyme, the cholesterol esters were quantitatively decomposed into free cholesterol and fatty acids. In the presence of peroxidation, the hydrogen peroxide generated reacted with 4-aminoantipyrine, forming a purple-bluish dye that was directly proportional to cholesterol concentration and was measured photometrically. All these parameters were measured using the Hitachi 717 automatic analyzer (Hitachi, Tokyo, Japan). The LDL-c concentrations were calculated from the Friedewald equation according to the manufacturer’s instructions [35]:

LDL-cholesterol: [LDL-c] = [C] − [HDL-c] − [TG/5](5)

### 3.4. Atherogenic Indices

After determining the concentration in mmol/L of total cholesterol (TC), total triglyceride (TG), HDL-c, and LDL-c fractions, atherogenic indices [AIP, CRR, AC, and cardioprotective indeks (CPI)] were calculated by using the values of lipid profile parameters in the following way:

Atherogenic index of plasma (AIP) = Log[TG/HDL-c](6)

Atherogenic coefficient (AC): AC = [TC − HDL-c/HDL-c](7)

Cardiac risk ratio (CRR): CRR = [TC/HDL-c](8)

Cardioprotective index (CPI): CPI= [HDL-c/LDL-c](9)

### 3.5. Statistical Analysis

Statistical analyses were performed using Statistica 13.0 software (Stat-Soft, Tulsa, USA). Enzyme activity was expressed as the mean value of the group (±SD of the mean). Multiple comparisons between the control and the treated groups were done by ANOVA and Tukey post hoc tests. The level of statistical significance was set at *p* ≤ 0.05 [36].

## 4. Results

### 4.1. Chemical Composition of Ethanolic Extract of Propolis

**Figure 1:**
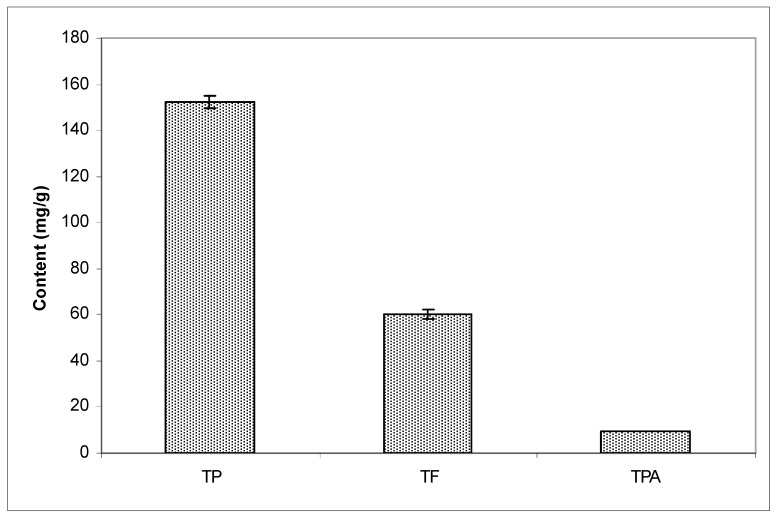
Content of total phenols (TP), total flavonoids (TF), and total phenolic acids (TPA) in ethanol extract of propolis.

Spectrophotometric and HPLC analysis confirmed that raw propolis was a poplar-chemotype. Spectrophotometric analysis showed that EEP contained TP of 152.33 ± 2.59, TF of 59.99 ± 2.04, and TPA of 9.68 ± 0.07 (Figure 1).

**Figure 2:**
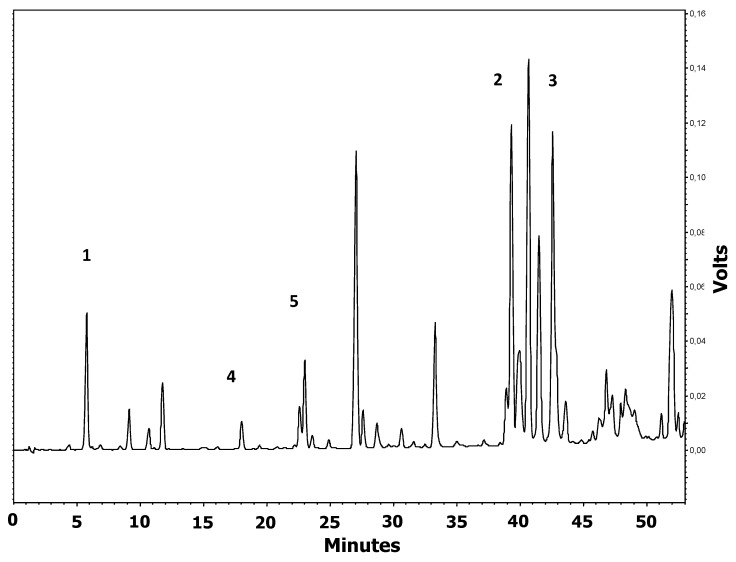
Chromatogram of ethanol extract of propolis used in the study monitored at 290 nm. Peaks are identified as: 1: caffeic acid; 2: chrysin; 3: galangin; 4: quercetin; 5: naringenin.

HPLC analysis of propolis showed the typical flavonoid-aglycones, including chrysin and galangin, as the most abundant polyphenols in EEP, naringenin, quercetin, as well as caffeic acid (Figure 2). The concentration of polyphenols in EEP was as follows: quercetin 0.28%, naringenin 0.63%, caffeic acid 1.32%, galangin 2.12%, and chrysin 2.45% (Figure 2).

### 4.2. β-Carotene Bleaching Assay

**Figure 3:**
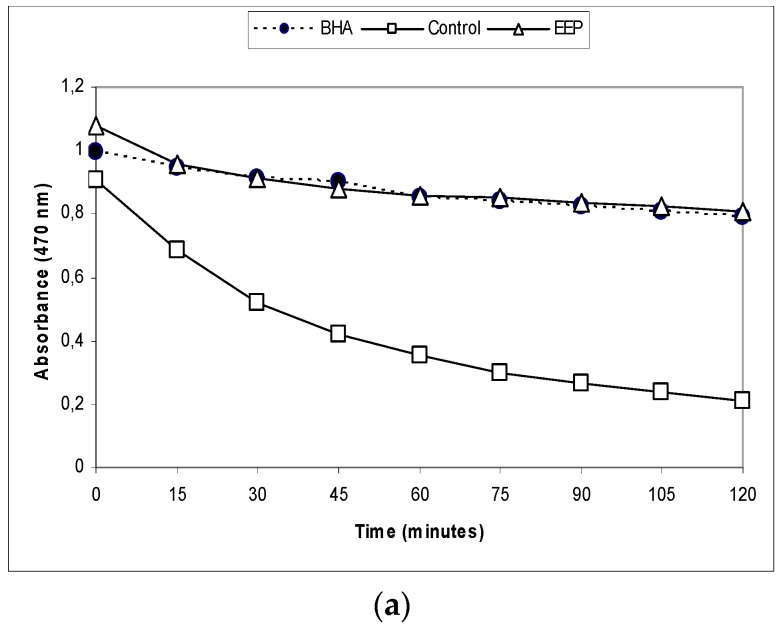

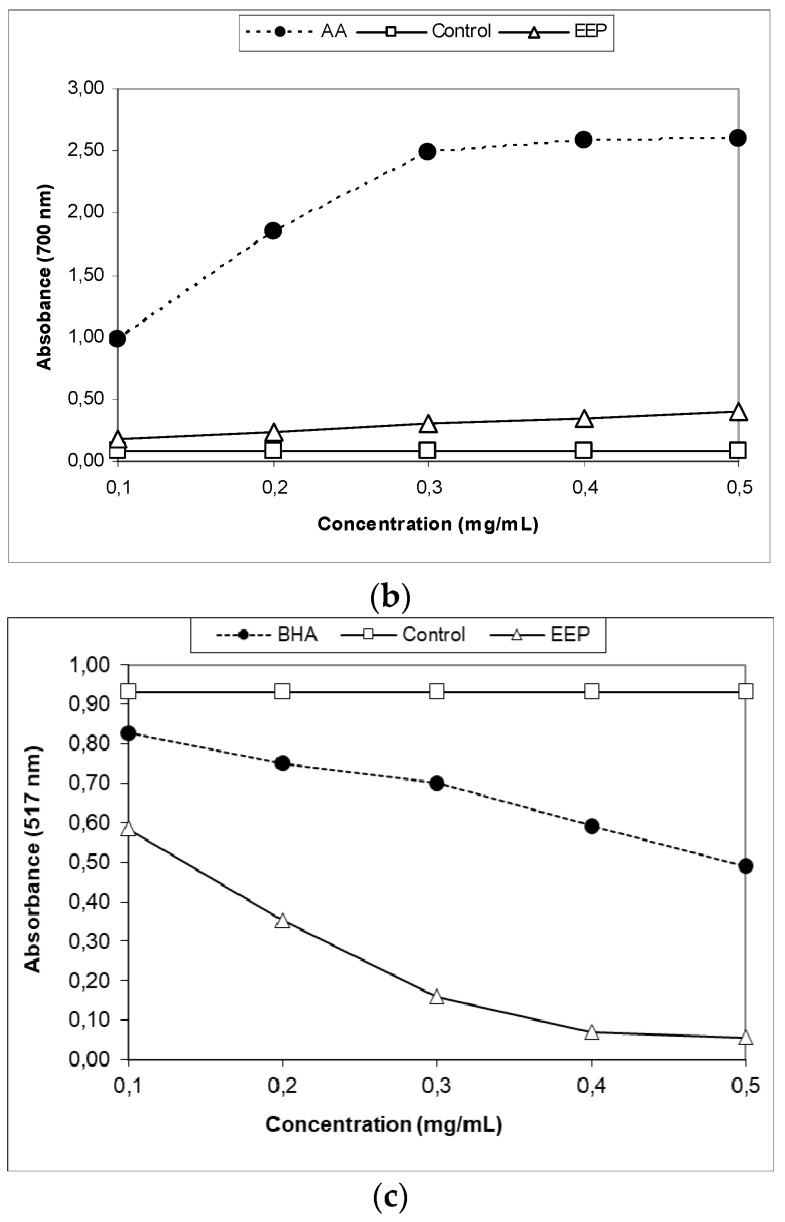
Antioxidative capacity of ethanol extract of propolis analyzed by three different methods: (**a**) β-carotene bleaching (BCB) assay, (**b**) the reducing power of the extracts, (**c**) 2,2-diphenyl-1-picrylhydrazyl radical-scavenging (DPPH) radical-scavenging activity. BHA: butylated hydroxy-anisole; AA: ascorbic acid; EEP: ethanol extract of propolis.

In the BCB assay, the oxidation of linoleic acid produced free radicals due to the removal of the hydrogen atom from diallylic methylene groups of linoleic acid [25]. The highly unsaturated β-carotene then was oxidized by the generated free radical. Degradation of the orange colored chromophore of β-carotene could be monitored spectrophotometrically. However, the presence of antioxidant constituents could prevent the bleaching of β-carotene because of their ability to neutralize the free radical. The reduction of absorbance of β-carotene-linoleic acid emulsion in the presence of the EEP is shown in Figure 3a.

In the β-carotene bleaching assay, the EEP were compared with a well-known antioxidant, BHA. The EEP showed almost the same antioxidant potential (AP) as BHA (87.30 ± 0.39 versus 91.67 ± 0.49) (Table 2).

### 4.3. The Reducing Power of the Ethanolic Extract of Propolis

The reducing power of the antioxidant activity measured the color change from yellow to green and blue depending on the EEP reduction power and was an indicator of the antioxidant activity. Figure 3b shows an increase in EEP concentration-dependent reduction ability; the increase was linear (*r*^2^ ≥ 0.94), while the absorption at 700 nm for the mixture with ascorbic acid remained fairly constant at higher concentrations (*r*^2^ = 0.81), reaching the maximum limit. Slopes of the trend lines (SRP) were calculated (Table 2) for the three lowest concentrations, where *r*^2^ > 0.95 for all the samples. The activity of EEP was a bit lower than the activity of ascorbic acid; RSP and EEP versus ascorbic acid was 0.56 ± 0.00 versus 7.53 ± 0.24 (Table 2). The reducing power of the ascorbic acid and the EEP increased with the concentration.

### 4.4. DPPH Radical-Scavenging Activity

The ability of antioxidants for DPPH radical scavenging is supposed to be due to their hydrogen donating property. This led to a decrease in absorbance in the solution with DPPH free radicals, which was measured spectrophotometrically at 517 nm. The scavenging effects of EEP on DPPH free radicals are shown in Figure 3c, while the EC_50_ value is shown in Table 2. At the applied concentrations, RSA of the samples and the standard increased linearly (*r*^2^ ≥ 0.99). The EEP showed strong DPPH free radical-scavenging activity (Figure 3c), but it was somewhat lower than the activity of BHA; RSA (EC_50_) EEP versus BHA was 27.82 ± 0.44 versus 6.02 ± 0.10.

### 4.5. Chelating Activity

Production of hydroxyl radicals occurs in the process known as Fenton’s reaction and is associated with excessive storage of iron or disorders in proteins involved in its transport or storage in numerous pathological conditions [3,20]. The chelating ability of the extracts toward ferrous ions was investigated, but in the investigated concentrations (0–1000 µg/mL), EEP did not show any chelating ability towards ferrous ion (Table 2).

### 4.6. Food Intake and Body Weight Gain

**Figure 4:**
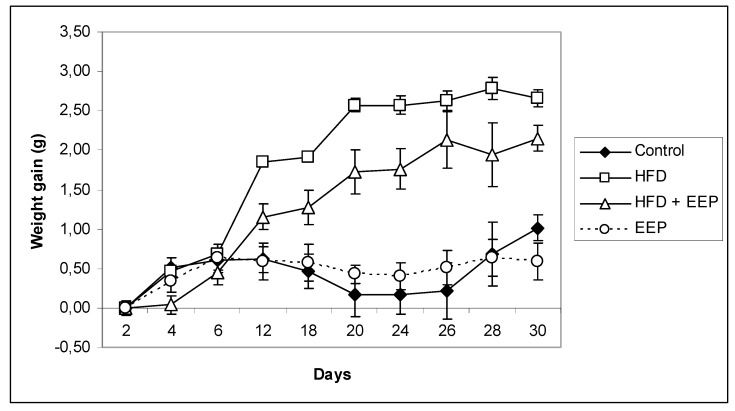
Effect of ethanol extract of propolis on weight gain in high-fat diet (HFD) fed mice. Mice were given HFD, HFD + EEP at dose 50 mg/kg or EEP (50 mg/kg) once daily for 30 days by a single intragastric application. Data are presented as the mean ± SD. Number of mice per groups: nine.

Administration of 50 mg/kg EEP that contained 152.33 ± 2.59 mg/g of total phenols (Figure 1) significantly reduced the weight gain compared to the control group and all other groups (Figure 4). Over the course of the experimental period, none of the experimental animals died, nor did they show abnormal food intake or behavioral changes. Comparison of the weight gain (Figure 4) showed that, until the 10th day, the weight did not differ significantly between the treated groups. At the end of the experimental period on the 30th day of the experiment, mice on a HFD had higher (*p* ≤ 0.05) weight gain than the control and all other experimental groups (Figure 4). The difference between the control and the HFD receiving groups started to become pronounced and significant from the 20th until the 30th day of the experiment. EEP applied together with the HFD reduced weight gain of the experimental animals (*p* ≤ 0.05). An important observation from the experimental results is that the weight gain between the control and the HFD + EEP groups became significantly different (*p* ≤ 0.05) between the 10th and the 20th day of treatment, and this difference contributed to the final difference among those groups and the HFD receiving group at the end of the experiment. The group that received EEP had approximately 33% lower weight gain than all other experimental animals (*p* ≤ 0.05) (Figure 4).

During the 30 consecutive feeding days, mice in the four groups showed an increase in body weight with different degrees (Table 3). After 30 days, the body weight gain in the HFD group was higher than that in the control group, indicating that HFD intake caused additional body weight gain and obesity in the HFD groups. The body weight gain in the HFD + EEP group was similar but a little lower than that in the HFD group, while weight gain in the EEP group was much lower. Additionally, intragastric application of the combination of the HFD + EEP treatment group reduced food intake compared to the control. However, the HFD group had significantly reduced (*p* < 0.05) food intake compared to control (Table 3). The HFD group of mice had a significantly greater food efficiency ratio (FER) in relation to the control group (0.92 ± 0.07 versus 0.30 ± 0.06). Supplementation of EEP decreased FER.

### 4.7. Serum Lipid Biochemical Parameters

The effect of EEP on serum compounds, liver triglycerides, and cholesterol in mice fed the HFD for 30 days is presented in Table 4 and shows that, in comparison to the control group, the groups receiving the HFD had significantly higher (*p* < 0.05) triglyceride levels in serum (39.8%) and liver tissue homogenate (44.1%) as well as cholesterol levels (31% in serum and 56.8% in liver tissue). In the group receiving the HFD supplemented with EEP, cholesterol levels were decreased in both serum and liver tissue (18.9% and 25.1%, respectively), and the triglyceride level in this group was significantly lower (11.4% in serum and 28.1% in liver) than in the group receiving only the HFD. Furthermore, the group receiving only EEP had significantly lowered triglyceride levels compared to all other groups (*p* < 0.05). Moreover, there was a slight decrease in the triglyceride and the cholesterol levels compared to the control group. The HDL cholesterol level was unaffected in all groups compared to the control group. The LDL levels in serum significantly increased in mice fed with the HFD (73.7%) and the HFD + EEP treated groups compared to the controls. However, the most significant difference between the two groups was that the LDL levels in groups supplemented with EEP were significantly lower (59.7%) than in the group receiving the HFD only.

### 4.8. Atherogenic Indices

**Figure 5:**
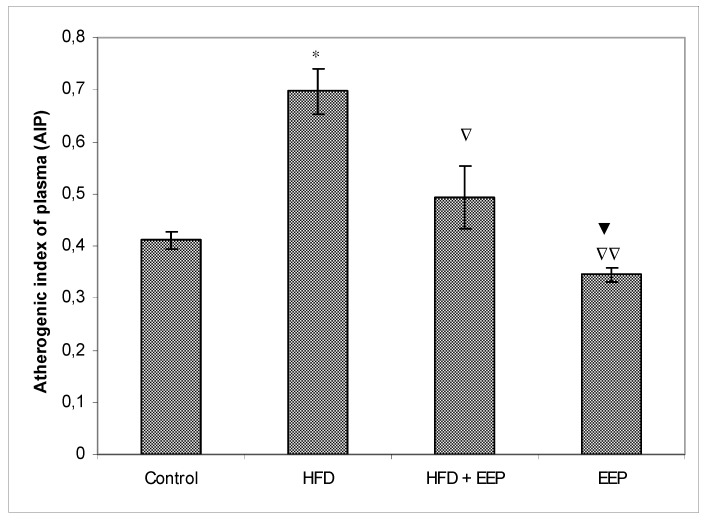
Effect of ethanol extract of propolis on atherogenic index of plasma (AIP). Mice were given HFD, HFD + EEP at a dose 50 mg/kg or EEP (50 mg/kg) once daily for 30 days by a single intragastric application. Atherogenic index of plasma was calculated according to the formula: AIP = Log [TG/HDL-c]. Number of mice per groups: nine. *: Significantly different from the control group (* *p* ≤ 0.05). ^∇^ Significantly different from the HFD group (^∇^ *p* ≤ 0.05; ^∇∇^ *p* ≤ 0.01).^▼^ Significantly different from the HFD + EEP group (^▼^ *p* ≤ 0.05). TG: total triglyceride.

**Figure 6:**
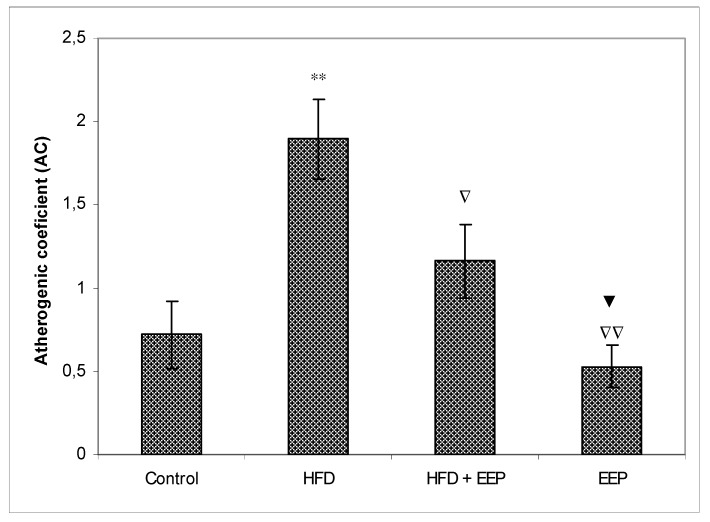
Effect of ethanol extract of propolis on atherogenic coefficient (AC). Mice were given HFD, HFD + EEP at a dose 50 mg/kg or EEP (50 mg/kg) once daily for 30 days by a single intragastric application. Atherogenic coefficient was calculated according to the formula: AC = [TC − HDL-c/HDL-c]. Number of mice per groups: nine. *: Significantly different from the control group (** *p* ≤ 0.01). ^∇^ Significantly different from the HFD group (^∇^ *p* ≤ 0.05; ^∇∇^ *p* ≤ 0.01). ^▼^ Significantly different from the HFD + EEP group (^▼^ *p* ≤ 0.05). TC: total cholesterol.

**Figure 7:**
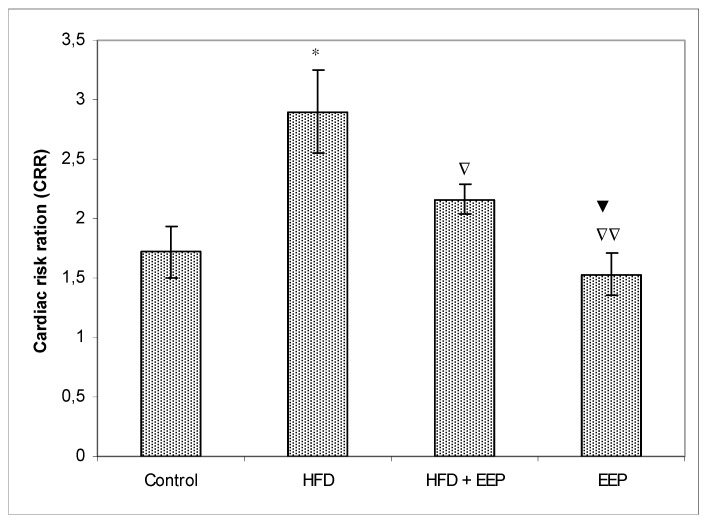
Effect of ethanol extract of propolis on cardiac risk ration (CRR). Mice were given HFD, HFD + EEP at a dose 50 mg/kg or EEP (50 mg/kg) once daily for 30 days by a single intragastric application. Cardiac risk ratio was calculated according to the formula: CRR = [TC/HDL-c]. Number of mice per groups: nine. * Significantly different from the control group (* *p* ≤ 0.05). ^∇^ Significantly different from the HFD group (^∇^ *p* ≤ 0.05; ^∇∇^ *p* ≤ 0.01). ^▼^ Significantly different from the HFD + EEP group (^▼^ *p* ≤ 0.05).

**Figure 8:**
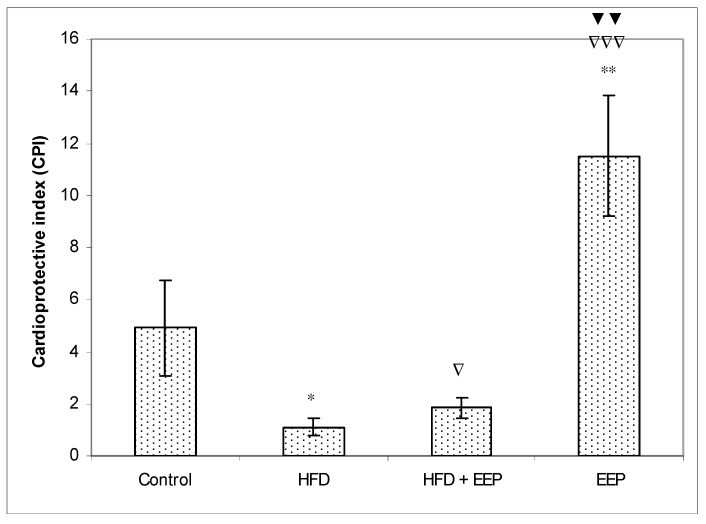
Effect of ethanol extract of propolis on cardioprotective index (CPI). Mice were given HFD, HFD + EEP at a dose 50 mg/kg or EEP (50 mg/kg) once daily for 30 days by a single intragastric application. Cardioprotective index was calculated according to the formula: CPI = [HDL-c/LDL-c]. Number of mice per groups: nine. * Significantly different from the control group (* *p* ≤ 0.05; ** *p* ≤ 0.01). ^∇^ Significantly different from the HFD group (^∇^ *p* ≤ 0.05; ^∇∇∇^ *p* ≤ 0.001). ^▼^ Significantly different from the HFD + EEP group (^▼▼^ *p* ≤ 0.01).

AIP, AC, and CRR (Figure 5, Figure 6 and Figure 7) were calculated and were significantly elevated in all groups receiving the HFD compared to the control, while CPI was decreased (Figure 8). The highest elevation of AIP among the treated groups was in the group receiving the HFD only. AIP between the HFD and the control was statistically significant (*p* < 0.05). AIP, AC, and CRR in the group receiving the HFD and EEP were also elevated compared to the control group but were significantly lower than those for the groups treated with the HFD only (*p* < 0.05). AIP for the EEP treated group remained at the level of the control group. The relationship value control versus EEP was 0.411 versus 0.344 (Figure 5). Treated mice with EEP showed no change (*p* > 0.05) in AC, AIP or CRR indices compared to the control group, except for CPI, where the EEP treated group increased CPI (*p* < 0.01) in relation to the control (control versus EEP group was 4905 versus 11512) (Figure 8).

### 4.9. Liver Enzyme Activity and Kidney Function

Metabolite profiles in serum (Table 5) show that the high-fat diet boosted the energy metabolism in the body. Total proteins, glucose, and urea were significantly higher (*p* < 0.05), and uric acid was significantly lower (*p* < 0.05) in the group receiving the HFD compared to the control group. The group receiving HFD + EEP had significantly elevated total protein, glucose, and uric acid concentration (*p* < 0.05), while urea was not significantly different compared to the control group. All values were significantly lower compared to the HFD treated group (*p* < 0.05). Treatment with EEP did not change any of the metabolic parameters monitored compared to the control group (*p* > 0.05). Glucose, total protein, and urea were significantly lower in the EEP treated group compared to the HFD and the HFD + EEP treated group (*p* < 0.05).

Serum alkaline phosphatase, aspartate aminotransferase, alanine aminotransferase, and lactate dehydrogenase activities (Table 6) in mice treated with the HFD were significantly higher than in all other groups (*p* < 0.05). Creatinine was the sole parameter that did not change due to treatment in any of the treated groups. The group treated with HFD + EEP showed an increase of enzymatic activity in the serum compared to the control group (*p* < 0.05), although all parameters were significantly lower than in the animals receiving the HFD only (*p* < 0.05). EEP reduced all measured parameters compared to all treated groups.

**Figure 9:**
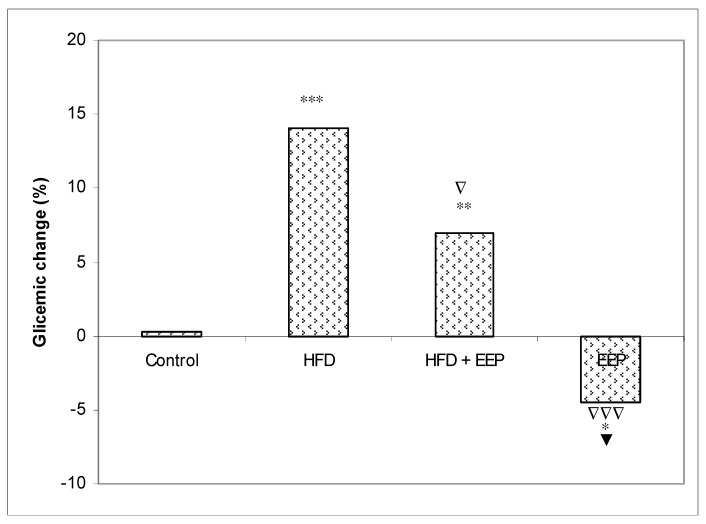
Effect of ethanol extract of propolis on percentage of glycemic change. Mice were given HFD, HFD + EEP at a dose 50 mg/kg or EEP (50 mg/kg) once daily for 30 days by a single intragastric application. The percentage of glycemic changes was calculated according to the formula: Glycemic changes (%) = (G_x_ − G_0_) / G_0_ × 100, where G_0_ = blood glucose level at 0 day and G_x_ = blood glucose level at 30 day. Number of mice per groups: nine. * Significantly different from the control group (* *p* ≤ 0.05; ** *p* ≤ 0.01; *** *p* ≤ 0.001). ^∇^ Significantly different from the HFD group (^∇^ *p* ≤ 0.05; ^∇∇∇^ *p* ≤ 0.001). ^▼^ Significantly different from the HFD + EEP group (^▼^ *p* ≤ 0.05).

A significant percent of the glycemic change was found as 14.11% (*p* < 0.001), 6.99% (*p* < 0.01), and −4.46% (*p* < 0.05) in mice treated with HFD, HFD + EEP, and EEP, respectively, compared to the control of 0.3 (Figure 9).

## 5. Discussion

Obesity is associated with metabolic disorders such as dyslipidemia, atherosclerosis, and type 2 diabetes and is the result of energy imbalance—an increased caloric intake ratio compared to calorie loss. The study of natural preparations with hypoglycemic, hypotensive, and hypolipidemic potential is an interesting approach in ethno-pharmacological research. The present study was designed to identify the antioxidative capacity of propolis and its long-term hypoglycemic and hypolipidemic effects as well as its weight improving pattern in mice fed with the HFD.

According to a paper by Martins and Redgrave [37], a diet-induced obesity animal model was developed to investigate human obesity. This model imitates the effect of human obesity better than the genetic obesity model and is based on a diet with a high fat content leading to obesity, hyperglycemia, and hyperlipidemia. In this work and other works, the elevated levels of TC, TG, and LDL-c in serum are factors of risk for the development of atherosclerosis and other cardiovascular diseases (CVD). In contrast, the elevated HDL-c level is associated with reduced risk for atherosclerosis.

Our results show that mice fed with the HFD and EEP over 30 days had higher levels of HDL-c compared with mice fed only with the HFD and consequently had lower levels of serum triglycerides and LDL-c, suggesting that EEP can effectively regulate metabolism triglycerides and cholesterol in HFD fed mice. Thus, propolis may be beneficial for treating patients with hypercholesterolemia and hypertriglyceridemia.

In this study, EEP increased plasma HDL cholesterol levels (Table 2) in both normolipidemic and hyperlipidemic mice. This effect may be mediated by the presented polyphenols/flavonoids in propolis [38]. However, coronary heart disease is accompanied and connected to high plasma LDL cholesterol [39]. Oxidation of LDL into oxidated low-density lipoprotein (ox-LDL) indicates the first step of atherosclerosis in cardiovascular diseases, stimulating the immune and the inflammatory reactions that initiate the process of atherosclerotic plaque buildup. Oxidized LDL damages vascular endothelial walls, induces the production of local hormones from blood vessel walls, promotes inflammation, and attracts macrophages that uptake ox-LDL via scavenger receptors and transform macrophages into foam cells. High HDL reverses cholesterol transport by scavenging excess cholesterol from peripheral tissues to the liver for its metabolism and excretion [40] and by inhibiting the oxidation of LDL. In addition, HDL can reduce or neutralize the atherogenic effects of oxidized LDL in artery walls. This effect may be increased by EEP and its antioxidative capacity (Figure 3). EEP increases HDL-c levels while reducing LDL-c and has the properties to reduce oxidation of LDL by two different antioxidant mechanisms—the activation of the transcription factor NrF2 and the enhancement of the antioxidant enzymes such as heme oxygenase-1, phase II detoxification enzymes, and enzymes involved in glutathione (GSH) metabolism [40,41,42,43]. The secondary mechanism could refer to the neutralization of oxidative species, and it inhibits the activation of the nuclear transcription factor-*κ*B (NF-*κ*B) signaling pathways. Reduced levels of LDL-c in HFD mice treated with EEP are the beneficial aspect of this current research in addition to showing the possible anti-atherosclerotic effects of EEP. This observation was consistent with the calculated indices such as AIP, AC, CRR, and CPI, which were all (exept CPI) attenuated following intervention with the EEP treatment (Figure 5, Figure 6, Figure 7 and Figure 8). These indices are valuable in assessing the risk of developing cardiovascular diseases; the more accurate the increase in AIP, AC, and CRR is, the more there is a predisposition for cardiovascular disease. CPI is highly improved in EEP treated normolipidemic and hyperlipidemic mice.

According to our results based on the antioxidative capacity of EEP, it seems that EEP with its compounds is a strong inhibitor of LDL oxidation. Oxidized LDL lipoproteins can be diminished with EEP by its antioxidant capacity, which can inactivate ROS and consequently counteract plasma LDL oxidation and reduce inflammation of the blood vessel endothelium. This antioxidative mechanism could contribute to the protective effect against cardiovascular diseases or other chronic diseases connected with oxidative stress. It seems that the consumption of the propolis diet was linked with hypolipidaemic effects specifically associated with decreased levels of LDL-c and TG and increased levels of HDL-c. EEP could exert health benefits not only by scavenging free radicals but also by hypoglycemic and regenerative effects. Blood glucose reduction may relate to the bioactive compounds of EEP and its protective effect on β-cells, which could enhance the production of insulin or enhance cellular sensitivity responses to insulin. The positive effect of EEP is also apparent in its hypoglycemic action (Figure 9). The hypoglycemic effect of EEP (Figure 9) may be due to: (i) an increase in glucose utilization by peripheral tissues; (ii) inhibiting the activity of enzymes included in cholesterol biosynthesis; (iii) decreased absorption of glucose in the intestine via the inhibition of the enzymatic activity of α-glucosidase; (iv) increased activity of the hepatic enzyme glucokinase, the markedly reduced enzymatic activities of hepatic glucose-6-phosphatase and phosphoenolpyruvate carboxykinase (PEPCK); or (v) the improvement in lipolysis by reducing the activity of hormone-sensitive lipase [2,44].

In addition, EEP improved the lipid profile of animals fed with the HFD diet and lowered their atherogenic indices closer to the levels of the control animals. A decrease in atherogenic indices is a positive physiological effect. The positive effects of propolis are induced by its bioactive components, especially the most explored polyphenol/flavonoid components such as epigallocatechin gallate, curcumin, reservatrol, quercetin, chrysin, naringenin, caffeic acid, caffeic acid phenethyl ester, proanthocyanidins, gallic acid, rosemary acid, isorhamnetin, camphorol, lutein, and pinocebrin [2,3,6,38,42,45,46,47].

Our results regarding HFD supplementation to the standard in an ad libitum diet are consistent with the data of other authors who used the same mice model [48,49,50]. In addition to the potential to prevent hyperlipidemia and influence lipid metabolism, EEP also affected the overall body weight of the animals (Figure 4). The results suggest that EEP is capable of reducing weight gain with no different food intake (Table 3). This data confirms the role of EEP in the regulation of carbohydrate and lipid metabolism. Thus, the physiological mechanism by which EEP prevents obesity in mice is activated by at least ten consecutive doses of EEP treatment. Confirmation of that physiological observation was found in the EEP treated group where, on the 10th day, the weight gain decreased in comparison to all other groups and remained rather uniformly unchanged until the end of the experimental period. To normalize weight gain change to food intake, we estimated the FER, which was calculated by dividing the weight increase by the food intake. In the HFD group, the FER was 3.1× greater in relation to the control group, which is a typical symptom of obesity, while in the HFD + EEP group, the FER was 2.3×. Food intake of these treatment groups was lower because dietary fat is a strong suppressor of food intake due to the fat induced secretion of satiety peptides. Our findings are similar to those in Hong et al. and Shin et al. [51,52]. These data collectively suggest that the supplementation of EEP attenuates HFD induced body weight gain, which is attributable to fat mass reduction possibly by inhibiting adipogenesis and increasing energy expenditure [51,52]. Further experiments will show what biochemical changes in lipid physiology occur during that period and whether they attribute to the overall body weight and lipid metabolism (Table 4). There are only a few works dealing with high EEP consumption and long-term consumption of this honeybee product [53].

Liver enzyme profiles, which are sometimes used as screening marker of hepatotoxicity, measured in the EEP treated group in this experiment remained similar to the control group, indicating that no unfavorable effects were noticed in the experimental animals. EEP showed a hepatoprotective role. It reduced the lipid content in the liver (Table 4) and improved the liver function, as indicated by liver enzymes such as aspartate aminotransferase (AST), alanine aminotransferase (ALT), alkaline phosphatase (ALP), and lactate dehydrogenase (LDH) in mice fed with the HFD and in healthy mice (Table 6). These enzymes are liver-specific enzymes that provide a deep insight about hepatic functioning; they elevate in cases of hepatic injuries or malfunctioning.

Renal function was also improved after EEP treatment in normolipidemic and hyperlipidemic mice (Table 5). Therefore, Oršolić et al. [3] demonstrated that the antioxidant and the anti-inflammatory effects of EEP were able to attenuate hypertension and structural hepatorenal damage in alloxan-induced diabetes mice models. Similar results were found in Koya-Miyata and co-workers that used Brazilian propolis, but there are differences between Croatian and Brazilian propolis [50,53]. The plant sources represent different ecosystems that have a great impact on the physical structure and the most important chemical composition of propolis, thus the chemical composition of propolis depends on the site of collection, th egeographic zone, the local flora, and the bee species [17,18,19,20]. Flavonoid-aglycones (flavones, flavonols, flavanones) and phenolic acids and their esters are the main biologically active components in propolis from the Northern Hemisphere, whereas in Brazilian propolis, the main biologically active components are prenylated phenylpropanoids (prenylated cinnamic acids and their esters), cinnamic acid derivatives, chromane, naphthalene, and anthracene derivatives, triterpens and steroids, and di- and sesquiterpenes. Also, in Europe, Northern America, and non-tropical regions in Asia, the main plant sources of propolis are cracks in the bark of trees and leaf buds from poplars (*Populus* spp., mainly from *P. nigra* L.), followed by birches (*Betula* spp.), elms (*Ulmus* spp.), pine trees (*Pinus* spp.), oaks (*Quercus* spp.), willows (*Salix* spp.), horse chestnut trees (*Aesculus hyppocastanum* L.), spruce (*Picea* spp.), and the ash (*Fraxinus* spp.). The Brazilian sources are *Baccharis* spp. (mainly *B. dracunculifolia*), but bees collect resins from other plant species as well, such as *Araucaria* spp., *Clusia* spp., *Eucalyptus* spp., *Vernonia* spp., *Diclenia* spp., *Hyptis* spp., *Myrcia* spp., *Schinus* spp., and *Weinmania* spp. Bankova [54] determined by plant origin recognizes several chemotypes, but if we compare Croatian and Brazilian propolis, then we distinguish poplar- and alecrim-chemotypes of propolis. As already mentioned, differences in the biological activity of propolis have been found in samples gathered from different races of honeybees. Therefore, it is very important to define the biological activity of propolis. Chemical analysis shows that Croatian propolis used in this study had a higher content of bioactive components than the ones described in references [3,4,5,6,7,8,9,10,11,16,17,18,19,20]. European propolis is known to have a high flavonoid content, often surpassing 20% in comparison to Brazilian propolis [55]. According to the results of Kosalec et al., the Croatian sample of propolis was richer in extractable substances than the Brazilian sample (27.53% and 13.15%, respectively) using ethanol. Additionally, Croatian ethanolic propolis extracts demonstrated more potent antibacterial, antitumor, antimetastatic, and immunomodulatory activity than Brazilian ethanolic propolis extracts [14,15,20]. It seems that bergamot, an endemic plant of the Calabria region (Italy), has a similar content of flavonoids as Croatian propolis and has an effect on weight gain, lipid clearance, and blood triglycerides [56].

Summarizing all the results, we can conclude that oral administration of EEP suppresses overall weight gain, reduces lipid profiles in serum and liver, increases hepatorenal function, and reduces atherogenic indices in mice fed with a HFD.

## 6. Conclusions

In conclusion, the results of the in vivo study show that propolis regulates serum and liver lipid profiles and decreases body weight gain in mice. EEP can significantly lower atherogenic indices (AIP, CRR, and AC) and increase CPI in animals fed with a HFD. In addition, propolis as a mixture of polyphenolic compounds possesses antioxidative properties and has positive effects on hepatorenal functions and the lipoprotein distribution. Thus, this gives hope that the use of propolis may be used to treat obese patients with hyperlipidemia.

## Figures and Tables

**Table 1:** Composition of standardized pellet mouse feed.

**Composition of Standardized Pellet Mouse Feed**		
wheat	wheetstrow, hazelnut skins	sodium chloride
maize	fish meal	whey powder
soybean dehulled	dicalcium phosphate	soybeen oil
corn gluten feed	calcium carbonate	yeasts
**Analytical Components and Supplements (Vitamins and Minerals)**		
12% moisture	E672 (vitamin A)	E3 (Co)
18.50% protein	E671 (vitamin E)	E4 (Cu)
3% fats	E1 (Fe)	E5 (Mn)
6% crude fibers, 7% crude ash	E2 (I)	E6 (Zn)

**Table 2:** Radical scavenging activity (RSA EC_50_), slope of reducing power trendline (SRP), activity in β-carotene-linoleate assay (ANT), and metal chelating activity (ChA EC_50_) of ethanol extract of propolis and standards.

Assay	Standard (STD)	Activity	
		EEP	STD
ANT (%)	BHA	87.3 ± 0.39 ***	91.67 ± 0.49
RSA EC_50_ (µg/mL)	BHA	27.82 ± 0.44 ***	6.02 ± 0.10
SRP (mL/mg)	Ascorbic acid	0.56 ± 0.00 ***	7.53 ± 0.24
ChA EC_50_ (µg/mL)	EDTA	n.d.	7.18 ± 0.10

Values are means ± SD (*n* = 3). n.d.: not detected; *** significantly different from the control group (*** *p* ≤ 0.001). BHA: butylated hydroxyanisole; EDTA: ethylenediaminetetraacetic acid; EEP: ethanol extract of propolis.

**Table 3:** Weight gain, food intake, food efficiency ratio (FER) in HFD fed mice.

Treatments ^1^	Weight Gain (g)	Food Intake (g/daily)	Food Efficiency Ratio (FER) *
Control	1.02 ± 0.36	3.44 ± 0.18	0.30 ± 0.06
HFD	2.66 ± 0.13	2.9 ± 0.09 *	0.92 ± 0.07 *
HFD + EEP	2.15 ± 0.36	3.1 ± 0.11	0.69 ± 0.03 *
EEP	0.59 ± 0.22	3.4 ± 0.4	0.17 ± 0.01 *^,∇,▼^

^1^ Mice were given HFD, HFD + EEP at a dose 50 mg/kg or EEP (50 mg/kg) once daily for 30 days by a single intragastric application. Number of mice per groups: nine. Data are presented as the mean ± SD. * Significantly different from the control group (* *p* ≤ 0.05). ^∇^ Significantly different from the HFD group (^∇^ *p* ≤ 0.05).^▼^ Significantly different from the HFD + EEP group (^▼^ *p* ≤ 0.05). * Food efficiency ratio: weight gain (g)/food intake (g/daily).

**Table 4:** Effects of ethanol extract of propolis on serum compounds, liver triglycerides, and cholesterol in high-fat diet fed mice.

Tissue	Parameters	Treatments ^1^			
		Control	HFD	HFD + EEP	EEP
Serum	HDL-c (mg/dL)	45.23 ± 3.19	38.94 ± 3.97	42.31 ± 3.49	41.56±2.51
	LDL-c (mg/dL) ^2^	9.22 ± 0.78	35.11 ± 0.75 *^,▼^	22.89 ± 0.86 *^,∇^	3.61±0.44 *^,∇,▼^
	TC (mg/dL)	77.79 ± 1.76	112.81 ± 1.85 *^,▼^	91.53 ± 1.63 *^,∇^	63.57±1.68 *^,∇,▼^
	TG (mg/dL)	116.66 ± 3.06	193.82 ± 2.87 *^,▼^	131.70 ± 2.81 *^,∇^	91.93±2.05 *^,∇,▼^
Liver	TC (mg/g)	7.73 ± 0.98	17.92 ± 1.12 *	13.43 ± 0.85 *	5.15±0.74 *^,∇,▼^
	TG (mg/g)	13.27 ± 2.37	23.74 ± 3.09 *^,▼^	18.45 ± 2.99 *^,∇^	9.69±1.44 *^,∇,▼^

^1^ Mice were given HFD, HFD + EEP at a dose 50 mg/kg or EEP (50 mg/kg) once daily for 30 days by a single intragastric application. Data are presented as the mean ± SD. Number of mice per groups: nine. ^2^ LDL-cholesterol: [LDL-c] = [C] − [HDL-c] − [TG/5] * Significantly different from the control group (* *p* ≤ 0.05). ^∇^ Significantly different from the HFD group (^∇^ *p* ≤ 0.05).^▼^ Significantly different from the HFD + EEP group (^▼^ *p* ≤ 0.05). HDL-c: high density lipoprotein cholesterol; LDL-c: low density lipoprotein cholesterol; TG: total triglyceride; TC: total cholesterol.

**Table 5:** Effect of ethanol extract of propolis on biochemical parameters in serum in high-fat diet fed mice.

Parameters	Treatments ^1^			
	Control	HFD	HFD + EEP	EEP
Creatinine (mg/dL)	0.47 ± 0.01	0.51 ± 0.04	0.47 ± 0.02	0.45 ± 0.01
Glucose (mg/dL)	82.9 ± 12.3	94.6 ± 10.2 *	88.7 ± 12.6 *	79.2 ± 11.7 ^∇,▼^
Total protein (g/L)	58.0 ± 0.61	74.5 ± 0.69 *^,▼^	65.7 ± 0.78 *^,∇^	50.7 ± 0.76 ^∇,▼^
Urea (mg/dL)	45.1 ± 2.07	51.9 ± 2.66 *^,▼^	47.9 ± 2.64 *^,∇^	42.2 ± 2.12 ^∇,▼^
Uric acid (mmol/L)	0.22 ± 0.01	0.16 ± 0.01 *^,▼^	0.32 ± 0.01 *^,∇^	0.20 ± 0.01 ^∇,▼^

^1^ Mice were given HFD, HFD + EEP at a dose 50 mg/kg or EEP (50 mg/kg) once daily for 30 days by a single intragastric application. Data are presented as the mean ± SD. Number of mice per groups: nine. * Significantly different from the control group (* *p* ≤ 0.05). ^∇^ Significantly different from the HFD group (^∇^ *p* ≤ 0.05). ^▼^ Significantly different from the HFD + EEP group (^▼^ *p* ≤ 0.05).

**Table 6:** Effect of ethanol extract of propolis on serum enzyme activities in high-fat diet fed mice.

Parameters	Units	Treatments ^1^			
		Control	HFD	HFD + EEP	EEP
AST	(U/L)	170.44 ± 16.09	288.07 ± 18.70 *^,▼^	232.04 ± 15.44 *	119.66 ± 15.95 *^,∇,▼^
ALT	(U/L)	85.81 ± 1.72	104.37 ± 3.07 *^,▼^	94.10 ± 2.95 *	71.96 ± 2.06 *^,∇,▼^
ALP	(U/L)	384.23 ± 18.11	491.34 ± 17.61 *^,▼^	409.99 ± 16.85 *	274.07 ± 16.44 *^,∇,▼^
LDH	(U/L)	1433.51 ± 83.50	2276.18 ± 89.08 *^,▼^	1863.71 ± 86.51 *	1094.76 ± 84.45 *^,∇,▼^

^1^ Mice were given HFD, HFD + EEP at a dose 50 mg/kg or EEP (50 mg/kg) once daily for 30 days by a single intragastric application. Data are presented as the mean ± SD. Number of mice per groups: nine. * Significantly different from the control group (* *p* ≤ 0.05). ^∇^ Significantly different from the HFD group (^∇^ *p* ≤ 0.05). ^▼^ Significantly different from the HFD + EEP group (^▼^ *p* ≤ 0.05). AST: aspartate aminotransferase; ALT: alanine aminotransferase; ALP: alkaline phosphatase; LDH: lactate dehydrogenase. U/L: units per litre, 1 U (μmol/min) is defined as the amount of the enzyme that catalyzes the conversion of one micromole of substrate per minute under the specified conditions of the assay method.

## Abbreviations and Acronyms

TG	total triglyceride
TC	total cholesterol
HDL-c	high density lipoprotein cholesterol
LDL-c	low density lipoprotein cholesterol
VLDL	very low density lipoprotein
CHD	coronary heart disease
ALP	alkaline phosphatase
AST	aspartate aminotransferase
ALT	alanine aminotransferase
LDH	lactate dehydrogenase
SOP	standard operating procedure
EEP	ethanol extract of propolis
